# Parametric study on the behavior of CFRP-strengthened reinforced concrete deep beams with cut circular web openings in shear spans

**DOI:** 10.1038/s41598-026-40071-y

**Published:** 2026-02-17

**Authors:** Eren Yagmur

**Affiliations:** Department of Civil Engineering, Faculty of Engineering, Abdullah Gül University, 38080 Kayseri, Turkey

**Keywords:** CFRP (carbon fiber-reinforced polymer), Deep beam, Web opening, FEM (finite element model), Engineering, Materials science

## Abstract

Web openings in reinforced concrete deep beams are often necessary for functional purposes but substantially reduce structural performance. Carbon fiber-reinforced polymer (CFRP) strengthening is commonly employed to mitigate these effects. Previous studies typically examined openings in regions without stirrups or assumed closed stirrup configurations, overlooking the frequent stirrup damage that occurs in practice due to the high shear reinforcement in deep beams. In this study, three specimens from a prior experimental program were modeled in ABAQUS, and the numerical results were validated against experimental data. Openings of varying diameters were introduced by cutting reinforcements, and the beams were subsequently strengthened with CFRP laminates, and a parametric study was conducted. Results showed that increasing opening diameter markedly reduces load-carrying capacity and energy absoption, while thicker CFRP laminates partially restore performance. For example, a 300 mm opening in a 500 mm high unstrengthened beam reduced load capacity by 56% and energy absorption by 87%. Even when the opening diameter was less than one-third of the beam height, 1.8 mm CFRP laminates provided only limited improvement. Deep beam performance was strongly influenced by web opening size, and the effectiveness of CFRP strengthening was limited when stirrup integrity was compromised.

## Introduction

Reinforced concrete (RC) deep beams are widely used in many engineering applications, including high-rise buildings, bridges, and industrial structures, due to their high shear capacity and efficient load-transfer mechanisms. In contemporary construction practice, web openings of various shapes and sizes are frequently introduced in deep beams to accommodate essential infrastructure services such as electrical installations, water supply lines, telecommunication networks, and gas pipelines^1,2^. These openings may be planned during the design and construction stages or introduced at later stages of the structure’s service life in response to changing functional requirements. In existing structures, such interventions are commonly carried out using core drilling techniques, which predominantly result in circular openings^3^.

While openings planned at the design stage can be adequately addressed through appropriate engineering measures, openings introduced after construction significantly disturb the natural stress flow within the beam and lead to stress concentrations along the boundaries of the opening. This disturbance triggers crack initiation and propagation under both service and ultimate loading conditions^2^. Such localized cracking reduces the stiffness of the structural member and consequently results in a reduction in its load-carrying capacity^4–17^. The severity of this adverse effect depends on several parameters, including the location, size, and symmetry of the opening.

Previous studies have demonstrated that the location of web openings plays a critical role in the structural response of deep beams. Openings located within the shear span have been reported to cause wider diagonal cracking and more pronounced strength degradation compared to those placed outside the shear span^18^. In contrast, openings positioned at midspan generally have a limited influence on the global load–deflection behavior; however, variations in crack patterns and failure mechanisms have been observed depending on the exact location of the opening^19^. Openings intersecting the primary load-transfer path have been shown to result in significant reductions in both load-carrying capacity and ductility^10,20^. Furthermore, symmetrically placed openings have been reported to be more detrimental than asymmetrically located ones, particularly when positioned near critical stress trajectories^21,22^.

In addition to opening location, the size of the opening has been identified as a key parameter influencing the performance of RC deep beams. Experimental studies on beams with single and multiple openings have shown that increasing the opening diameter leads to pronounced reductions in ultimate load capacity and toughness, while ductility appears to be less sensitive to this parameter^23^. For beams containing multiple circular openings, the opening diameter has been reported to have a more dominant influence on structural performance than the shear span length^24^. Similarly, Ali and Saeed^25^reported a notable decrease in shear strength and overall structural efficiency with increasing opening size.

Considering that reinforced concrete structures are generally designed to maintain adequate performance over several decades^26,27^, mitigating the adverse effects of post-construction modifications is of paramount importance. To this end, various strengthening techniques have been proposed to control crack development and prevent premature failure^28–33^. Among these techniques, carbon fiber–reinforced polymer (CFRP) systems have attracted significant attention due to their high strength-to-weight ratio, corrosion resistance, and ease of application.

CFRP composites are high-performance materials produced by impregnating woven carbon fibers with a resin matrix. While carbon fibers provide high tensile strength, stiffness, and excellent fatigue resistance, the resin matrix protects the fibers against environmental effects such as moisture and ultraviolet radiation. Owing to their low density and high strength-to-weight ratio, CFRP composites enable the development of lighter and more efficient structural systems compared to conventional materials such as concrete and steel^34–36^. Moreover, their high resistance to corrosion and environmental degradation makes CFRP composites particularly suitable for strengthening applications in aggressive and harsh service environments^37^.

Experimental studies have demonstrated that CFRP strengthening can significantly enhance the structural performance of RC deep beams with web openings. For example, CFRP strengthening has been reported to increase the stiffness of beams with large circular and square openings by up to 33% and 17%, respectively^17^. Wrapping square openings with CFRP strips or sheets has resulted in an increase in load-carrying capacity of approximately 54%^8^, whereas similar strengthening applied to beams with large circular openings has led to an increase of about 15.32% in ultimate load capacity^38^. Other studies have also confirmed that CFRP laminates improve the initial cracking load, ultimate strength, and crack distribution^5,13,39^.

The strengthening of RC deep beams requires special consideration because their structural behavior deviates from classical flexural theory, and load transfer occurs predominantly through discontinuity regions (D-regions). ACI 318 − 19^40^defines deep beams as members for which the plane-sections assumption is not valid and recommends the use of the strut-and-tie model for their analysis and evaluation. Similarly, Eurocode 2 (EN 1992-1-1:2004)^41^states that linear elastic design approaches are not appropriate for deep beams and other D-regions and emphasizes that load transfer should be represented through compression struts and tension ties. Both design codes highlight the necessity of verifying the compressive capacity of load–support nodal regions, particularly in cases involving web openings or interruptions to the primary load-transfer path. In this context, clearly identifying the influence of post-installed openings on the load-transfer mechanism of RC deep beams and systematically evaluating this behavior within existing design frameworks are essential for the reliable assessment of strengthening effectiveness.

Although the literature on RC deep beams with web openings is extensive, most existing studies focus on beams with predesigned openings, where the openings are either located in regions without stirrups or formed by arranging closed stirrup configurations above and below the opening. In contrast, in practical rehabilitation applications, circular openings are often created by cutting through both the web reinforcement and the stirrups. Such interventions unintentionally compromise the structural integrity and significantly alter the internal force-transfer mechanism. Consequently, the structural behavior of deep beams under such realistic opening conditions has not been sufficiently addressed in the existing literature.

Moreover, although CFRP strengthening has been widely investigated, no systematic parametric study is available in the literature that examines the combined influence of opening size and CFRP laminate thickness on RC deep beams containing post-drilled circular openings formed by cutting reinforcement. This research gap is particularly pronounced for symmetrically placed openings that intersect critical stress-flow paths.

Given the high cost and practical limitations associated with experimental testing, finite element analysis has become an effective and widely accepted tool for investigating complex structural behavior. In the present study, the ABAQUS finite element software is employed to examine the structural performance of RC deep beams containing symmetrically placed circular web openings formed by cutting both longitudinal reinforcement and stirrups. The numerical models are first validated against experimental results reported by Jasim et al.^39^. Subsequently, a comprehensive parametric study is conducted to evaluate the effectiveness of CFRP strengthening with varying laminate thicknesses for beams with different opening sizes. The findings of this study are intended to contribute to a better understanding of the structural behavior of CFRP-strengthened RC deep beams with post-installed web openings and to serve as a reference for future research in this field.

## Materials and methods

**Fig. 1:**
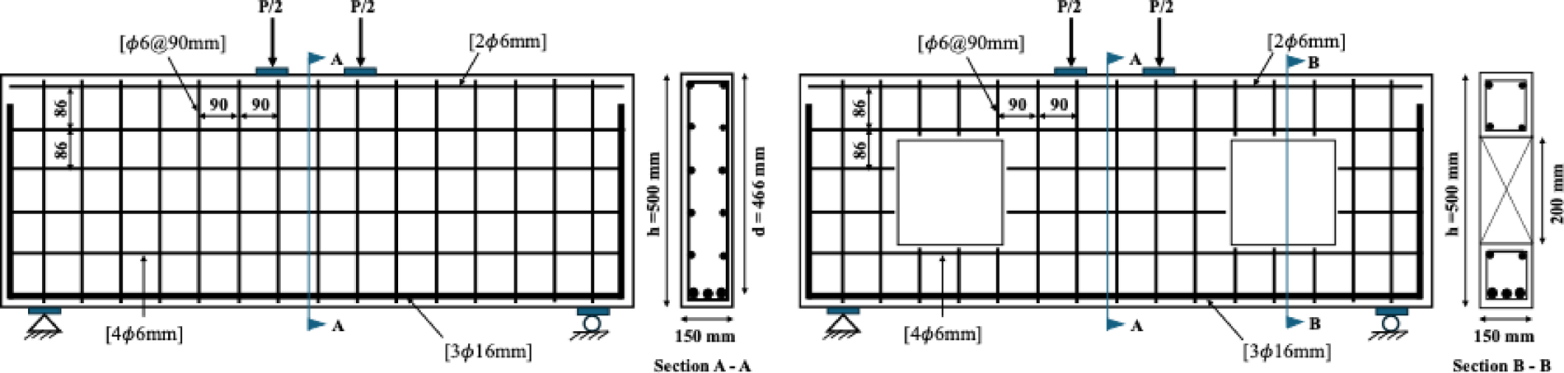
Geometric and reinforcement details of beams without and with web openings.

In the present study, the beam configuration proposed by Jasim et al.^39^was adopted to investigate the effect of openings on the structural performance of deep beams (Fig. 1). Three specimens from the previous study were selected for this purpose: a beam without an opening (DP-S1), a beam with a symmetrical square opening of 200 × 200 mm (DP-S1-C-O1-WS), and a beam strengthened with CFRP around the opening (DP-S1-C-O1-S). All specimens had a length of 1500 mm, a cross-sectional dimension of 150 × 500 mm, and a shear span-to-depth ratio of 1.1. The general reinforcement layout applied to all beams is presented in Fig. 1, while the mechanical properties of concrete and steel are summarized in Tables 1 and 2.

**Table 1:** Mechanical properties of the reinforcements.

Reinforcement	Measured diameter (mm)	Yield stress, $\:{f}_{y}$ (MPa)	Ultimate stress, $\:{f}_{u}$ (MPa)	Elongation (%)
*ϕ*6	5.5	623.96	685.5	8.5
*ϕ*16	16	569.67	668.79	12.5

**Table 2:** Concrete properties.

Compressive strength, $\:{{f}_{c}}^{{\prime\:}}$ (MPa)	Tensile strength, $\:{f}_{t}$ (MPa)	Rupture stress, $\:{f}_{r}$ (MPa)	Elastic modulus, $\:{E}_{c}$ (MPa)	Poisson’s ratio $\:\upsilon\:$
27	3.1	3.5	24,667	0.2

The experimental specimens were modeled and analyzed using the ABAQUS finite element program, and the numerical results were validated through comparison with the experimental data. The close agreement observed between the numerical simulations and the experimental results confirmed the reliability of the developed model for subsequent parametric studies. Previous investigations on deep beams with web openings have reported that the most critical structural behavior occurs in beams with symmetrical web openings^21,22^. Accordingly, a symmetrical opening configuration was adopted in the present study.

However, in practice, design conditions related to web openings cannot always be satisfied due to service requirements, architectural constraints, or post-construction interventions. Therefore, the main objective of the present study is to investigate the effects of web openings with the most unfavorable (critical) sizes and locations on the behavior of reinforced concrete deep beams. According to the literature, web openings with diameters of 150 mm and above are not recommended for use in deep beams without special reinforcement detailing or alternative strengthening techniques^42^.

On the other hand, the diameters of service ducts used in buildings may vary from relatively small sizes to values reaching 300–400 mm, depending on functional requirements. In this study, the overall depth of the beam specimens was selected as 500 mm. Considering this geometry, it was anticipated that the use of a 400 mm diameter opening would result in insufficient concrete sections above and below the opening to ensure the continuity of the compression strut. Therefore, the upper limit of the opening diameter was set to 300 mm.

Within this framework, four opening diameters—150 mm, 200 mm, 250 mm, and 300 mm—were considered to evaluate the influence of opening size on beam behavior. It was considered that successive increases in opening diameter would provide a systematic and convenient approach for assessing the effects of opening size on the internal force transfer mechanism and the overall structural response of the member.

Each beam configuration was subsequently strengthened using CFRP laminates with thicknesses of 1.0 mm, 1.4 mm, and 1.8 mm, and the numerical analyses were repeated for each strengthening scenario. The results obtained from the strengthened and unstrengthened configurations were then compared to evaluate the effectiveness of CFRP strengthening in improving the load transfer mechanism and enhancing the structural behavior of reinforced concrete deep beams with large web openings.

## Numerical model

### Model description

**Fig. 2:**
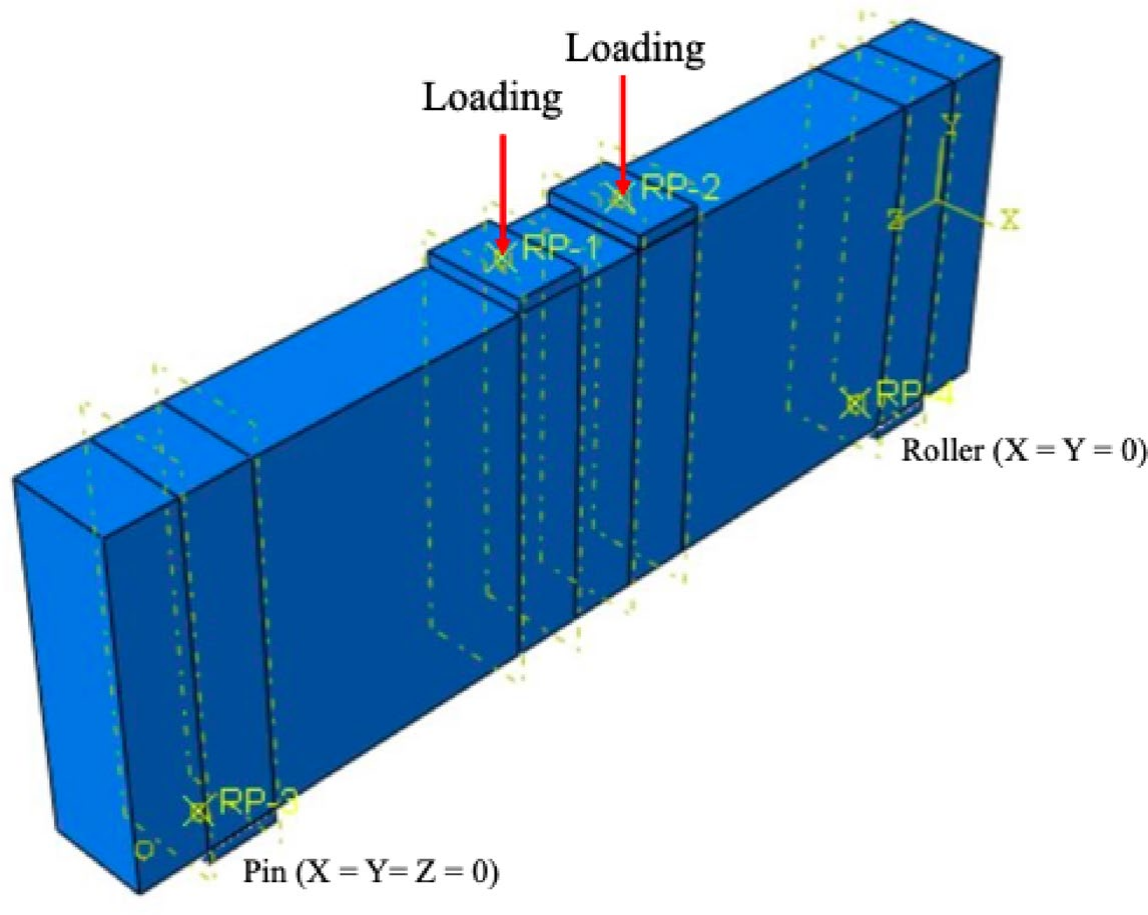
Modeling of loading and boundary conditions.

The experimental specimens investigated by Jasim et al.^39^were modeled using full-scale finite element models (FEMs). The specimens were represented as simply supported beams with pin-roller supports (Fig. 2). The pin support was fully constrained in all directions (X, Y, and Z), whereas the roller support allowed translation only in the vertical direction. To prevent potential damage to the concrete at both the loading and support points due to the monotonic displacement protocol applied during the static analysis, 20 mm-high and 100 mm-wide steel plates were incorporated. These plates were modeled as discrete rigid elements and were connected to the concrete using tie constraints.

Realistic behavior in FEM applications critically depends on accurate material modeling. To simulate the degradation of elastic stiffness in concrete resulting from both tensile and compressive plastic strains, established structural models from the literature were employed. Among these, the Concrete Damage Plasticity (CDP) model^43,44^implemented in ABAQUS is widely used. The CDP parameters adopted in this study are presented in Table 3.

**Table 3:** CDP parameters^39^.

Parameter	Elongation (%)
The dilation angle, $\:\psi\:$	36^o^
The eccentricity of the plastic potential surface, $\:e$	0.1
The ratio of biaxial to the uniaxial compressive yield stress, $\:{f}_{b0}/{f}_{c0}$	0.16
The ratio of the second stress invariant on the tensile meridian to that on the compressive meridian, $\:k$	0.667
The viscosity parameter, $\:\mu\:$	0.00001

The compressive behavior of concrete was described using the model developed by Saenz^45^, while the tensile response was represented using the model proposed by Nayal and Rasheed^46^and subsequently refined by Wahalathantri et al.^47^to prevent sudden deformations. This approach has been successfully applied in previous studies and has proven effective in accurately predicting the behavior of concrete specimens^39,48,49^.

The steel reinforcement was modeled as bilinear isotropic, following the approach of Jasim et al.^39^. The material properties required for this representation are provided in Table 1. Additionally, the modulus of elasticity and Poisson’s ratio of steel were assumed to be 200 GPa and 0.3, respectively. A perfect bond between concrete and reinforcement was assumed, and the interaction was modeled using an embedment constraint.

The CFRP material was modeled as linear elastic isotropic. Although unidirectional CFRP is inherently orthotropic, its behavior under load is dominated by the modulus along the fiber direction; thus, isotropic modeling is an acceptable approximation^50^. Brittle failure was assumed to occur once the material reached its ultimate tensile strength^51^. Previous studies on the interaction between CFRP and concrete have demonstrated that this modeling approach provides reliable predictions^50–52^. Accordingly, the CFRP laminates in this study were connected to the concrete using tie constraints to prevent shear failure.

A sensitivity analysis indicated that a 25 mm mesh was sufficient to capture the load-displacement response of the specimens. Although a 20 mm mesh is commonly used in similar studies^3,39,53^, it has been shown that 20 mm and 25 mm mesh sizes do not significantly affect the load-displacement curve results^54,55^. Considering the computational efficiency, a 25 mm mesh size was adopted for all models.

### Model verification

**Fig. 3:**
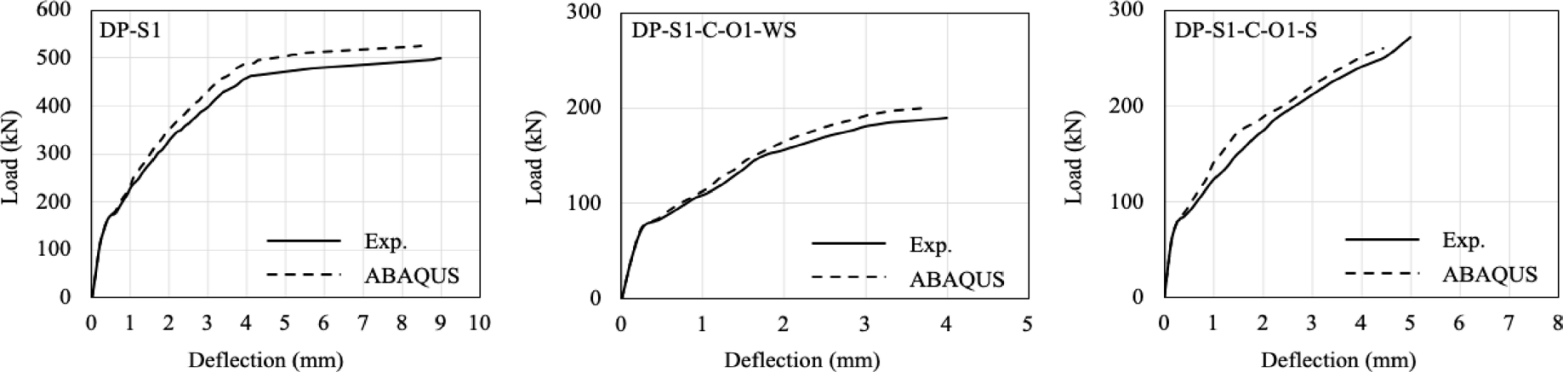
Comparison of the experimental and numerical results.

**Fig. 4:**
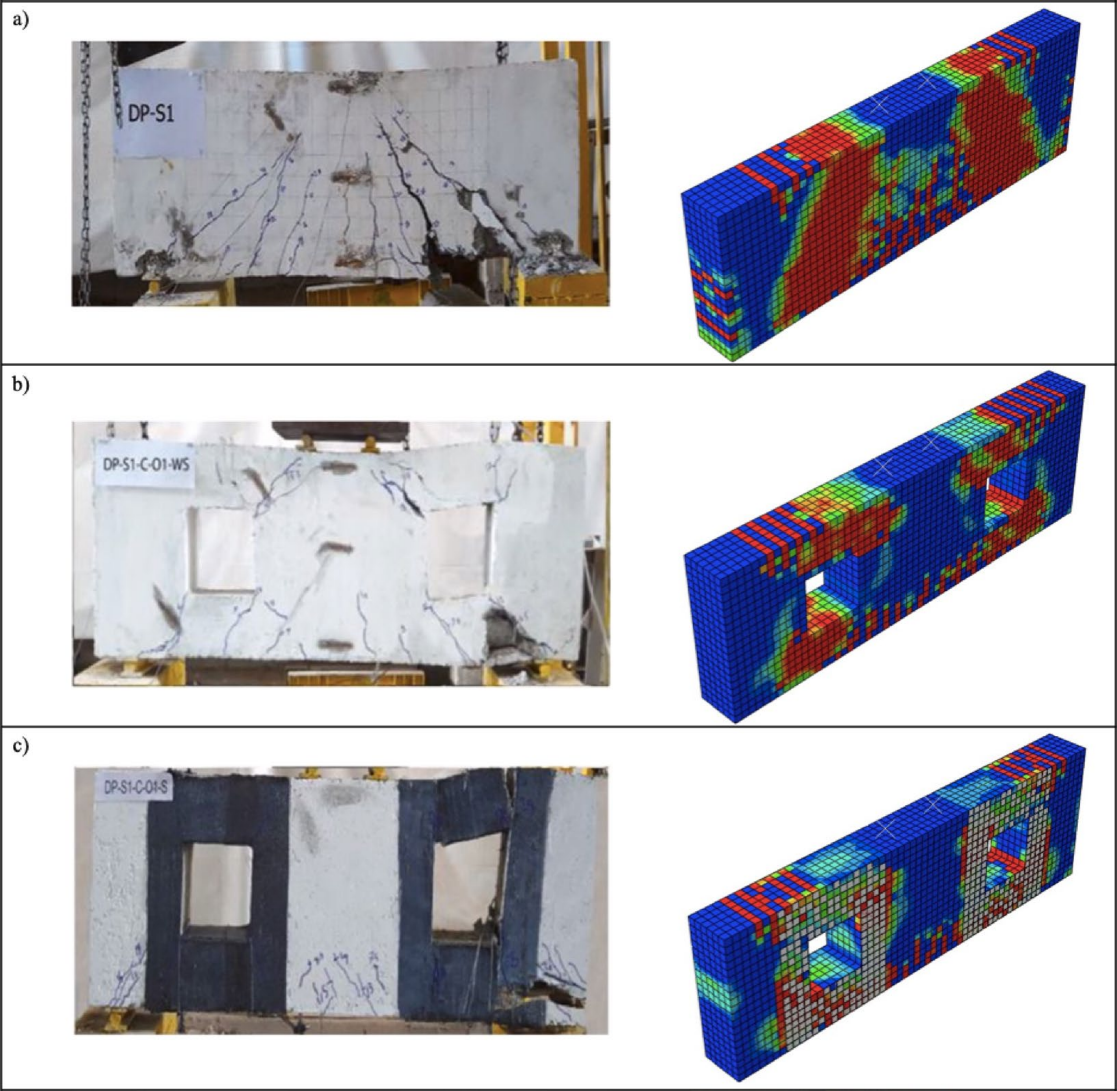
Experimental vs. numerical failure patterns: (**a**) DP-S11, (**b**) DP-S1-C-O1-WS, and (**c**) DP-S1-C-O1-S.

The load–displacement responses and crack patterns obtained from the finite element analyses (FEA) were compared with the experimental results, as shown in Figs. 3 and 4. Evaluation of the results in terms of load–displacement capacity and potential crack formation patterns confirmed the applicability of FEA for parametric studies.

**Fig. 5:**
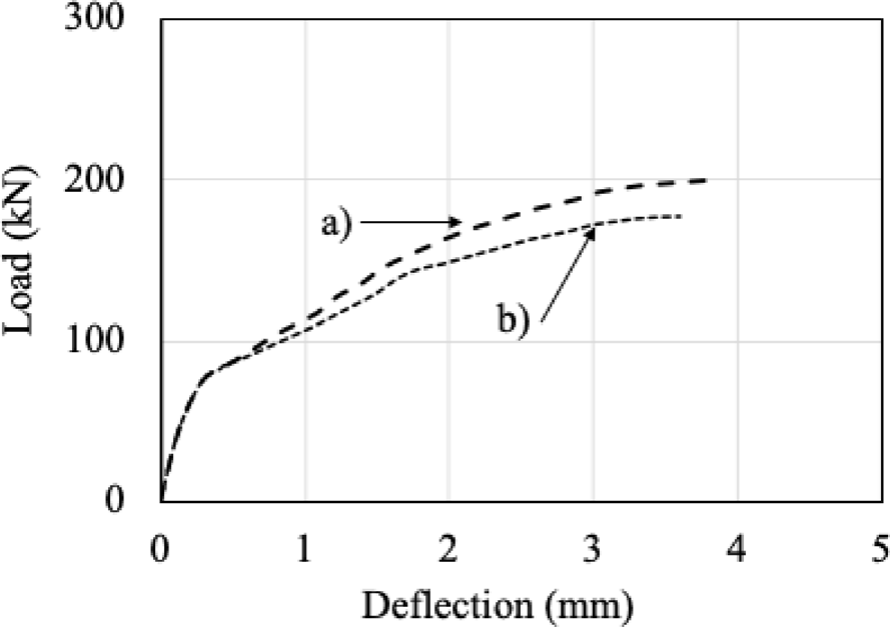
Comparison of the numerical results: (**a**) FEM of DP-S1-C-O1-WS with closed-end stirrup; (**b**) FEM of DP-S1-C-O1-WS with open-ended stirrup.

**Fig. 6:**
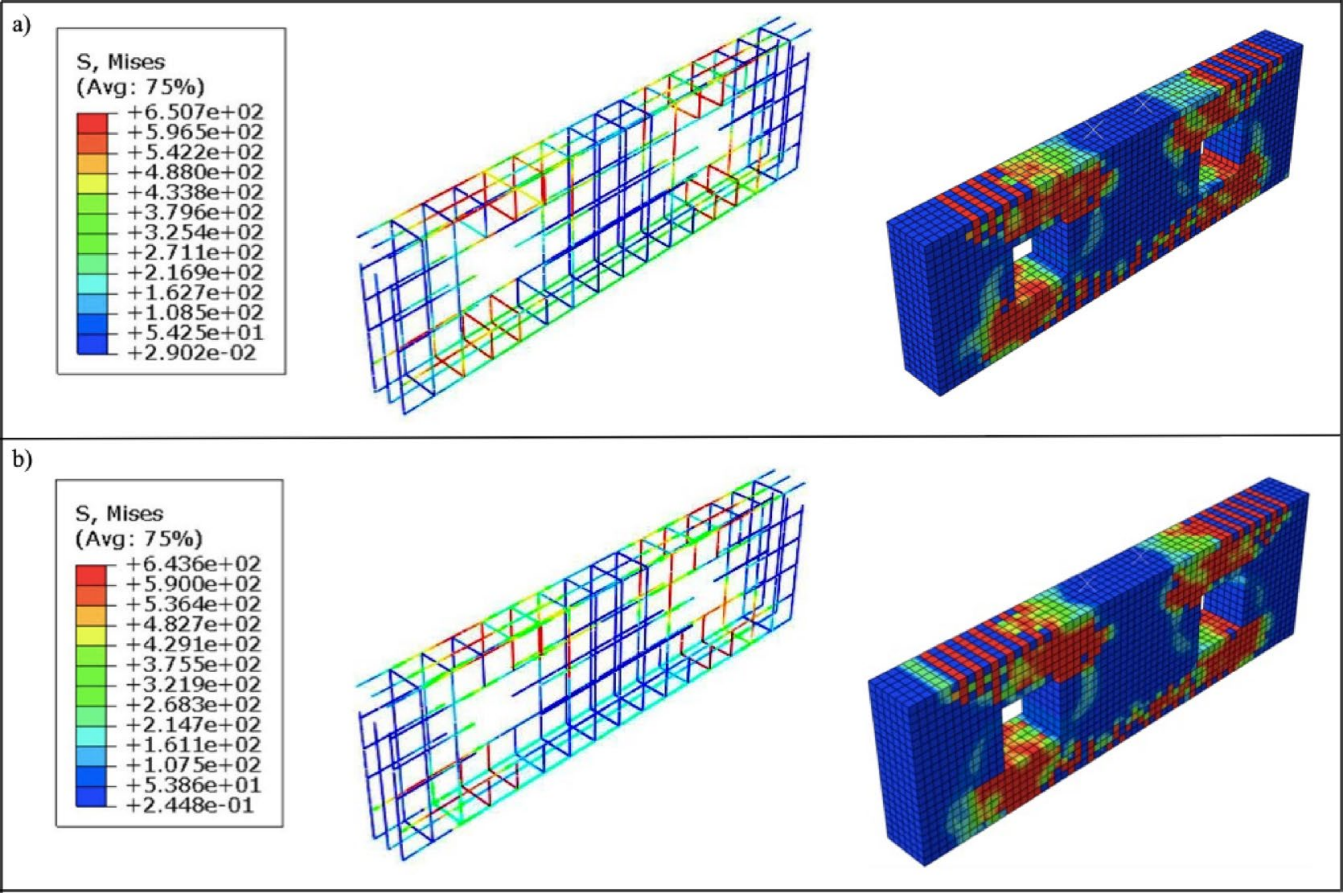
Comparison of the numerical failure patterns: (**a**) FEM of DP-S1-C-O1-WS with closed-end stirrup; (**b**) FEM of DP-S1-C-O1-WS with open-ended stirrup.

In the experimental program, stirrups around the openings of the specimens with openings were provided as closed (DP-S1-C-O1-WS and DP-S1-C-O1-S). Accordingly, all stirrups were modeled as closed during the numerical simulation of the experimental specimens. However, in practice, the stirrups are cut at the openings created later. To investigate the effects of closed versus open-ended stirrups on the specimen performance, the DP-S1-C-O1-WS specimen was remodeled with open-ended stirrups and analyzed, as illustrated in Figs. 5 and 6.

The load–displacement relationships obtained for both configurations are presented in Fig. 5. For the specimen with closed stirrups, the maximum displacement and load were 3.78 mm and 200.22 kN, respectively, whereas for the specimen with open-ended stirrups, these values were 3.59 mm and 177.09 kN. These results indicate that cutting the stirrups reduces the displacement by approximately 5% and decreases the load-carrying capacity by 11.55%.

Figure 6 presents the distribution of Von Mises stress and concrete tensile damage (DAMAGET), where red regions indicate tensile damage. The analysis reveals that closed stirrups result in higher reinforcement stresses and fewer cracks. In contrast, open-ended stirrups lead to increased cracking, particularly above the spans, due to the reduced confinement contribution of the stirrups. Moreover, a slight reduction in crushing zones at the specimen corners was observed when open-ended stirrups were used.

## Parametric study

**Fig. 7:**
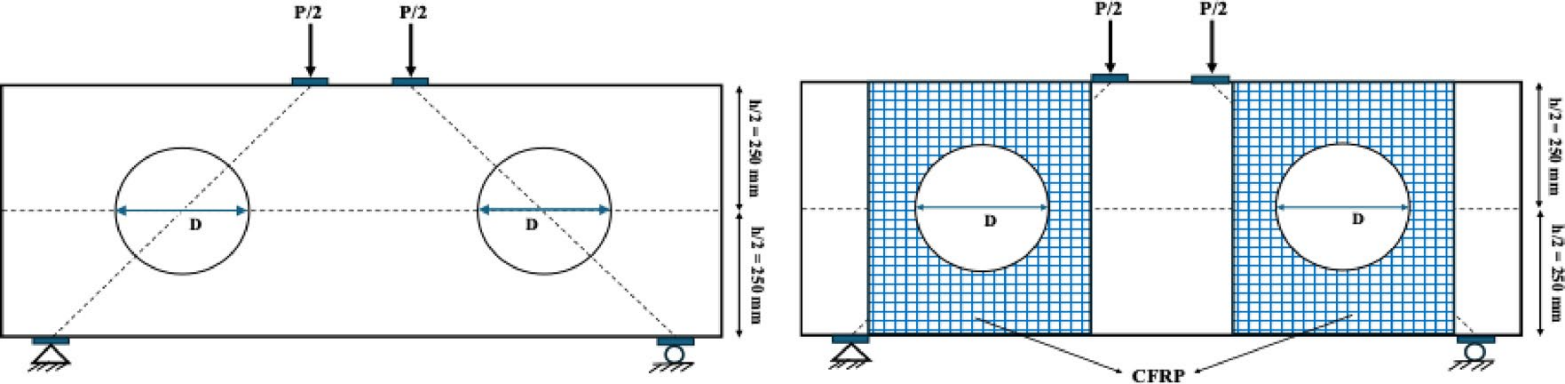
Schematic of the locations and strengthening of web openings.

**Fig. 8:**
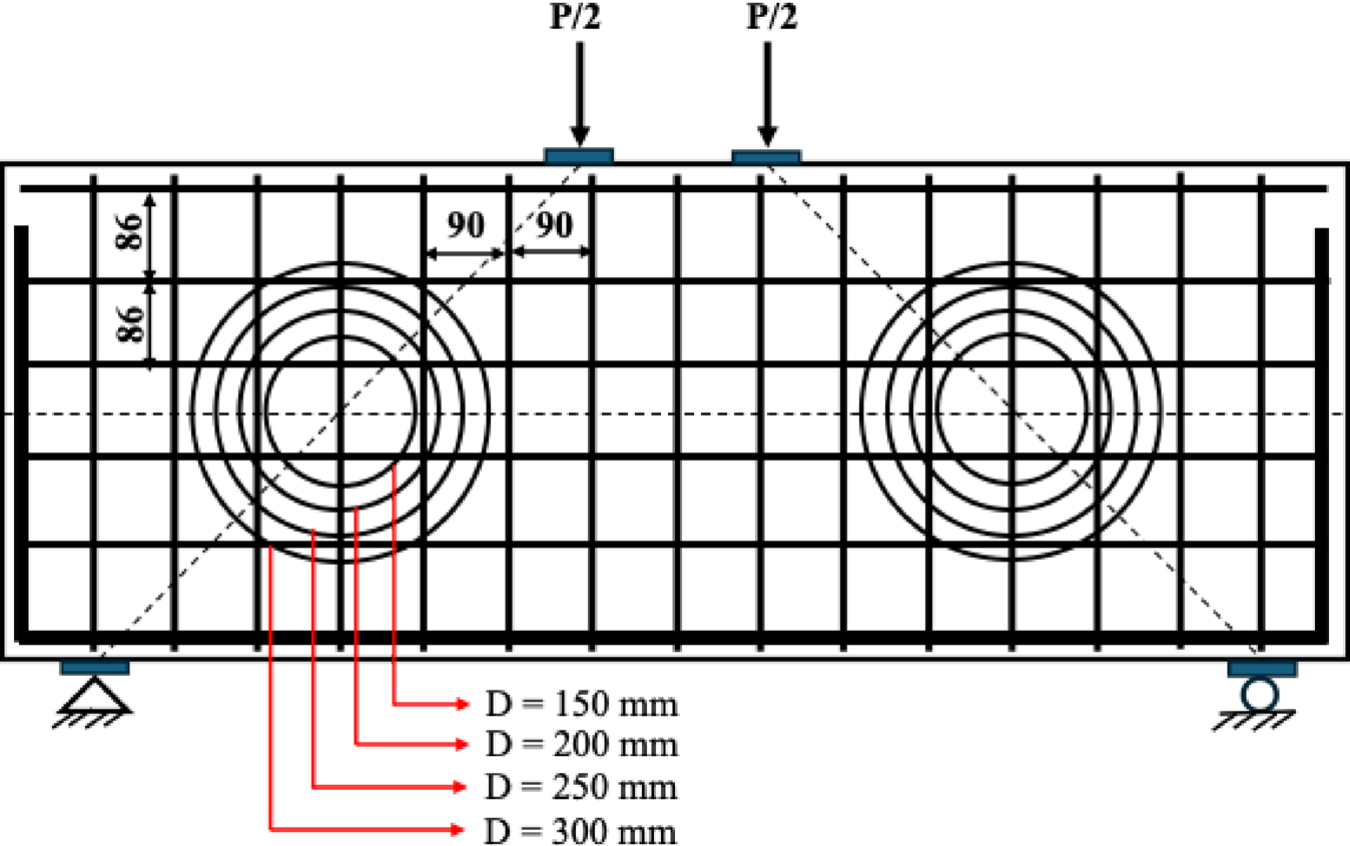
Schematic showing the reinforcement bars that need to be cut to create the web openings.

After validating the finite element models (FEMs), a parametric study was conducted to investigate the effects of opening size and the strengthening of beam specimens with CFRP laminates of varying thicknesses (1.0 mm, 1.4 mm, and 1.8 mm) on the structural behavior of deep beams with openings created by cutting the reinforcements. Previous experimental studies have indicated that the most critical effects among openings located at different positions occur when they are placed in the shear path^10,18,19^and symmetrically positioned^21,22^. Therefore, as illustrated in Fig. 7, symmetrical openings with diameters (D) of 150 mm, 200 mm, 250 mm, and 300 mm were introduced in both cut locations, and these specimens were subsequently strengthened with CFRP laminates of different thicknesses. All other parameters, including material properties, reinforcement configuration, and geometric dimensions, were kept identical to those reported by Jasim et al.^39^. Figure 8 depicts the reinforcement bars that must be cut to create the openings. Excluding the control specimen (DP-S1), a total of sixteen finite element models were developed, allowing for cross-comparisons to evaluate the effects of both opening size and CFRP thickness. To facilitate systematic analysis, the specimens in the parametric study were named according to their opening diameter and CFRP thickness. For instance, specimens with a 150 mm opening, unstrengthened and strengthened with 1.0 mm CFRP, are denoted as D150-0.0 and D150-1.0, respectively. This naming convention enables clear identification of each configuration and supports detailed comparative evaluation of the structural responses.

## Results and discussions

**Fig. 9:**
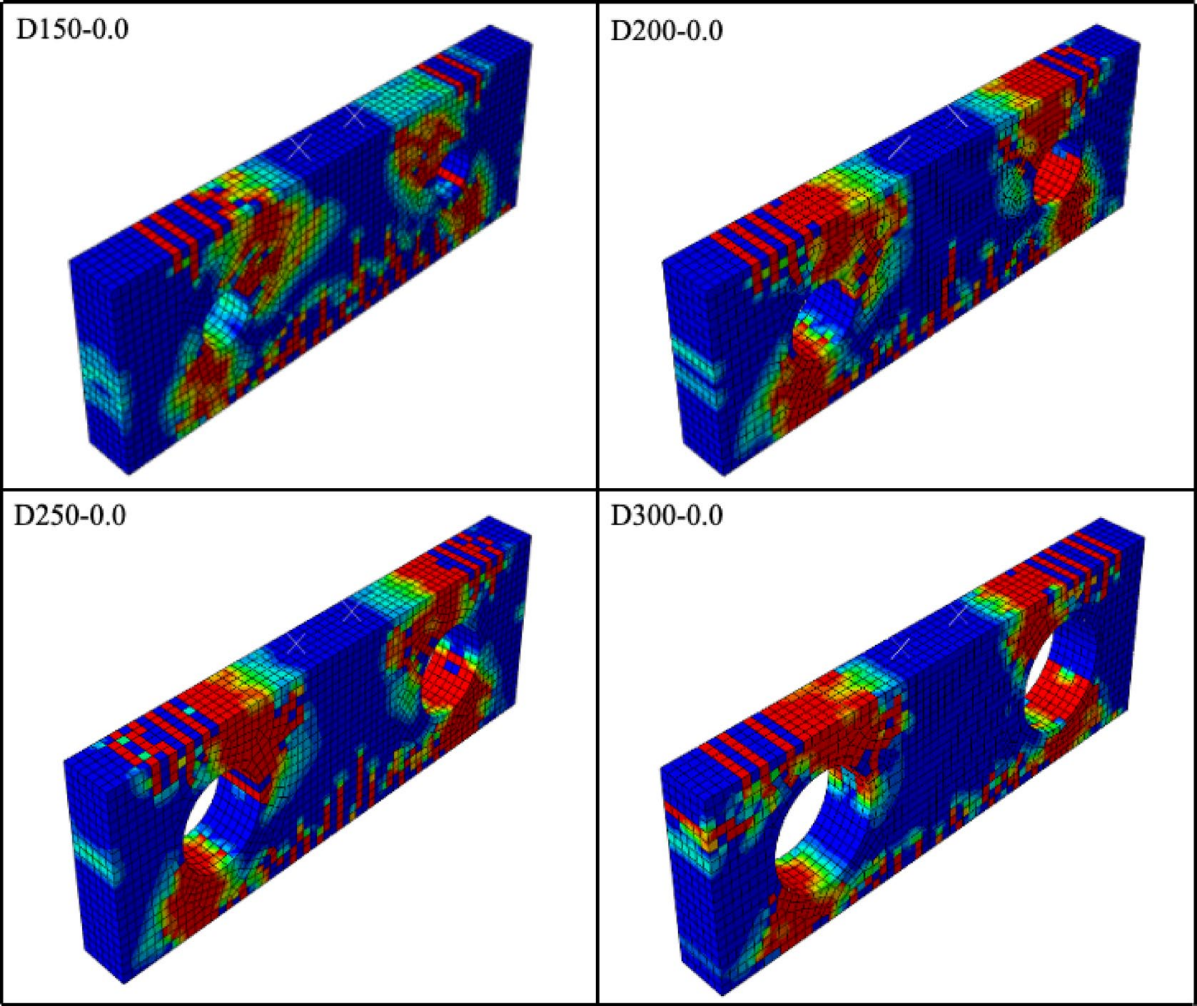
Damage patterns for unstrengthened FEMs.

The influence of web opening size on the structural performance of reinforced concrete specimens was first evaluated by comparing unstrengthened specimens from four series, namely D150, D200, D250, and D300. The corresponding damage patterns are presented in Fig. 9. In specimen D150-0.0, a dominant diagonal crack developed, extending from the support region toward the load application point. With increasing opening diameter, the observed damage mode shifted toward shear–compression failure in the chord near the loading point. This behavior can be attributed to the combined effects of a reduced stirrup ratio and a decrease in the effective concrete cross-section caused by the enlargement of the opening.

Web openings located along the strut flow significantly alter the internal force transfer mechanism of reinforced concrete deep beams and promote the occurrence of shear–compression failure, particularly in the regions above

**Fig. 10:**
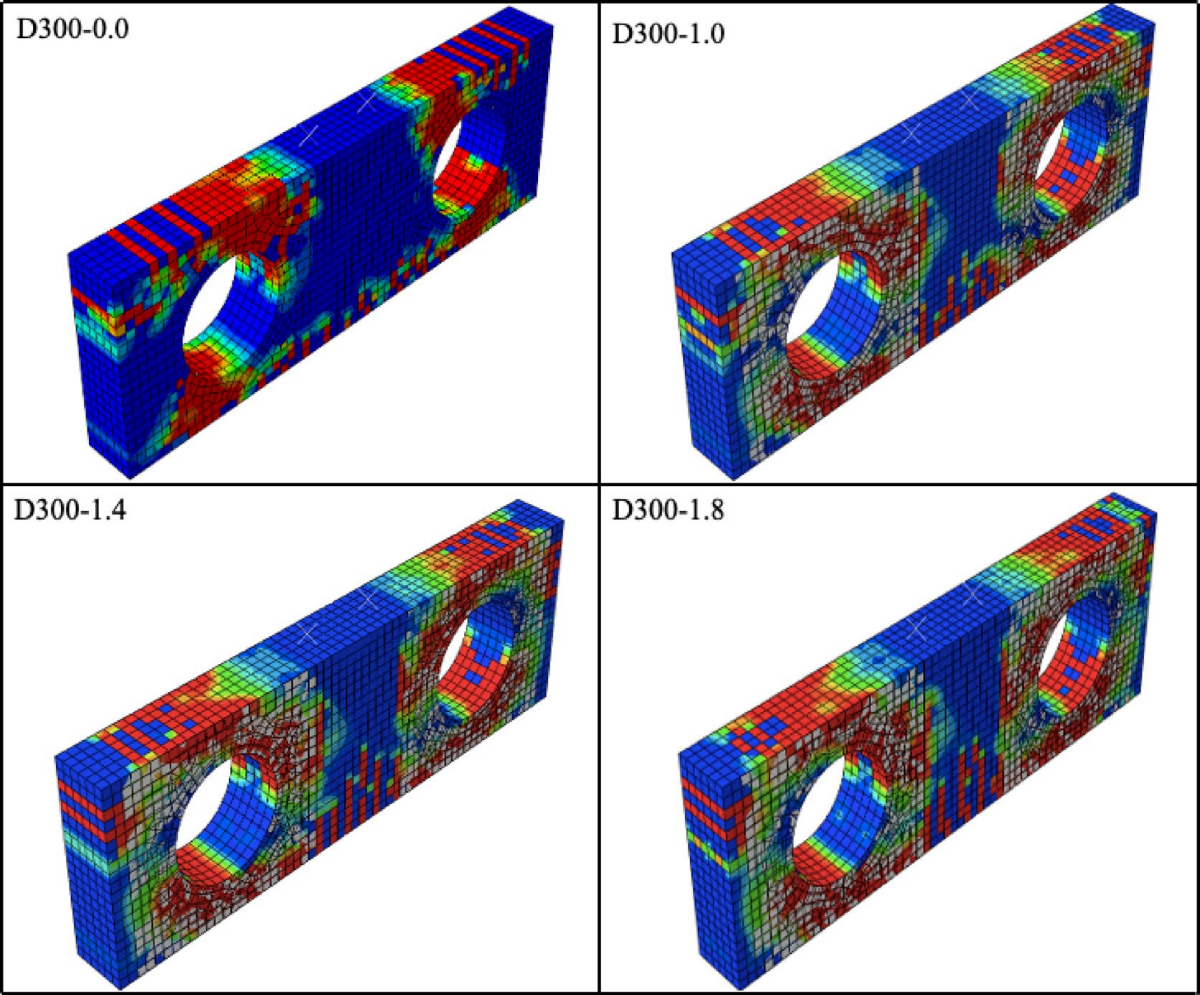
Damage patterns for strengthened FEMs of the D300 series.

and below the opening. In deep beams, load transfer is governed primarily by diagonal compression struts developing between the load and support regions, rather than by classical flexural behavior. When a web opening is positioned along this strut flow, the effective cross-sectional area of the diagonal compression strut is reduced, forcing the load path to deviate toward the upper and lower edges of the opening and resulting in pronounced compressive stress concentrations in these regions. Under increasing shear forces, these stress concentrations initiate diagonal cracking at the corners of the opening, with cracks propagating along the direction of the compression strut. As crack propagation progresses, the compressive load-carrying capacity of the concrete is gradually diminished, ultimately leading to localized concrete crushing in the regions above and below the opening. This failure mode typically occurs in a sudden and brittle manner without significant yielding of the reinforcement and is governed by parameters such as the size and location of the opening relative to the strut flow, the shear span-to-effective depth ratio, the amount and detailing of transverse reinforcement, and the compressive strength of the concrete.

Among the CFRP-strengthened specimens investigated in this study, the D300-0.0 specimen, which has the largest web opening diameter and therefore represents the most critical configuration, was selected as a representative example to more clearly illustrate the influence of the opening and the effectiveness of the strengthening mechanism. For this specimen, the DAMAGET distributions obtained from the analyses performed after strengthening with CFRP laminates of 1.0, 1.4, and 1.8 mm thickness are presented in Fig. 10.

In contrast to the pronounced narrowing of the compression strut observed in the unstrengthened specimen, the CFRP-strengthened configurations exhibit a more distributed stress pattern in the upper and lower chord regions around the opening, indicating a reorganization of the internal force transfer mechanism. This behavior suggests a transition from a single, constricted compression strut to a load transfer mechanism in which the forces are more effectively shared along the upper and lower chords, approaching a two-branch strut behavior. Moreover, increasing the CFRP laminate thickness enhances this effect, leading to a more stable and balanced shear–compression response of the specimen.

**Fig. 11:**
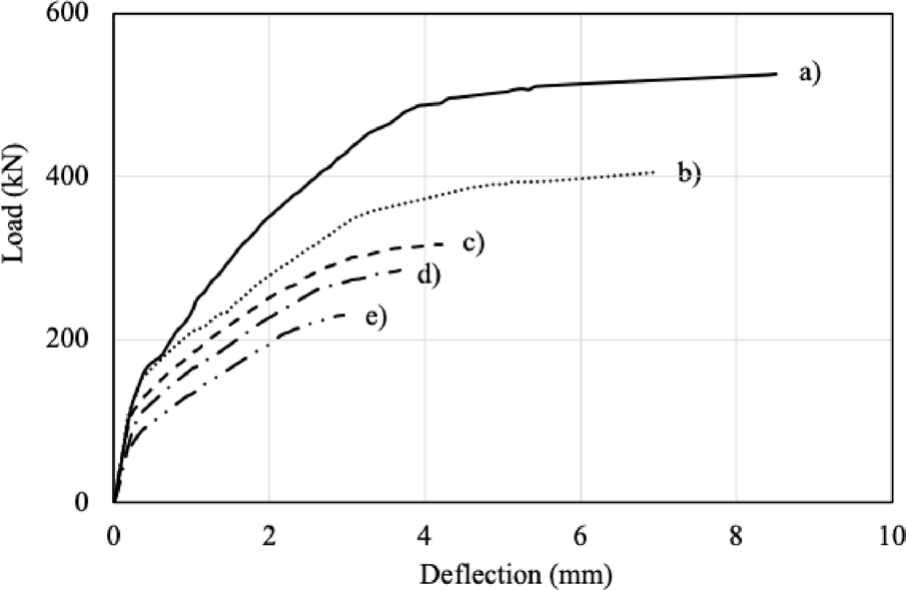
Comparison of load-displacement curves for unstrengthened elements: (**a**) DP-S_FEM, (**b**) D150-0.0, (**c**) D200-0.0, (**d**) D250-0.0, (**e**) D300-0.0.

The finite element model (FEM) analysis of the test specimen DP-S1, denoted as DP-S1_FEM, was employed as a control. Figure 11 compares the load-displacement responses of the unstrengthened specimens with DP-S1_FEM. Maximum load ($\:{P}_{U}$), maximum displacement ($\:{\varDelta\:}_{U}$​), normalized load ($\:{P}_{N}$), energy absorption capacity ($\:EAC$), and normalized energy absorption capacity ($\:{EAC}_{N}$​) are summarized in Table 4. The energy absorption capacity was determined by calculating the area under the load–displacement curve. The calculations were performed using numerical integration based on the trapezoidal rule (Eq. 1).

1 $$\:EAC=\sum\:_{i=1}^{n-1}\frac{{P}_{i}+{P}_{i+1}}{2}\left({\varDelta\:}_{i+1}-{\varDelta\:}_{i}\right)$$

Here, $\:{P}_{i}$​ and $\:{P}_{i+1}$​ represent successive load values, and $\:{\varDelta\:}_{i}$​ and $\:{\varDelta\:}_{i+1}$​ correspond to the displacements associated with these load values, $\:n$ is the total number of discrete load–displacement data points. The normalized load was calculated as the ratio of the maximum load of each specimen to that of DP-S1_FEM. Normalized energy absorption was determined as the ratio of the energy consumption of each specimen to that of DP-S1_FEM. As shown in Fig. 11, both the maximum load capacity and energy absorption decreased with increasing opening size. Specifically, the maximum load capacity decreased by 23%, 40%, 46%, and 56% for openings of 150, 200, 250, and 300 mm, respectively, whereas the energy absorption capacity decreased by 27%, 48%, 56%, and 87%. These results indicate that web openings within the shear span significantly compromise the structural performance of the specimens.

**Fig. 12:**
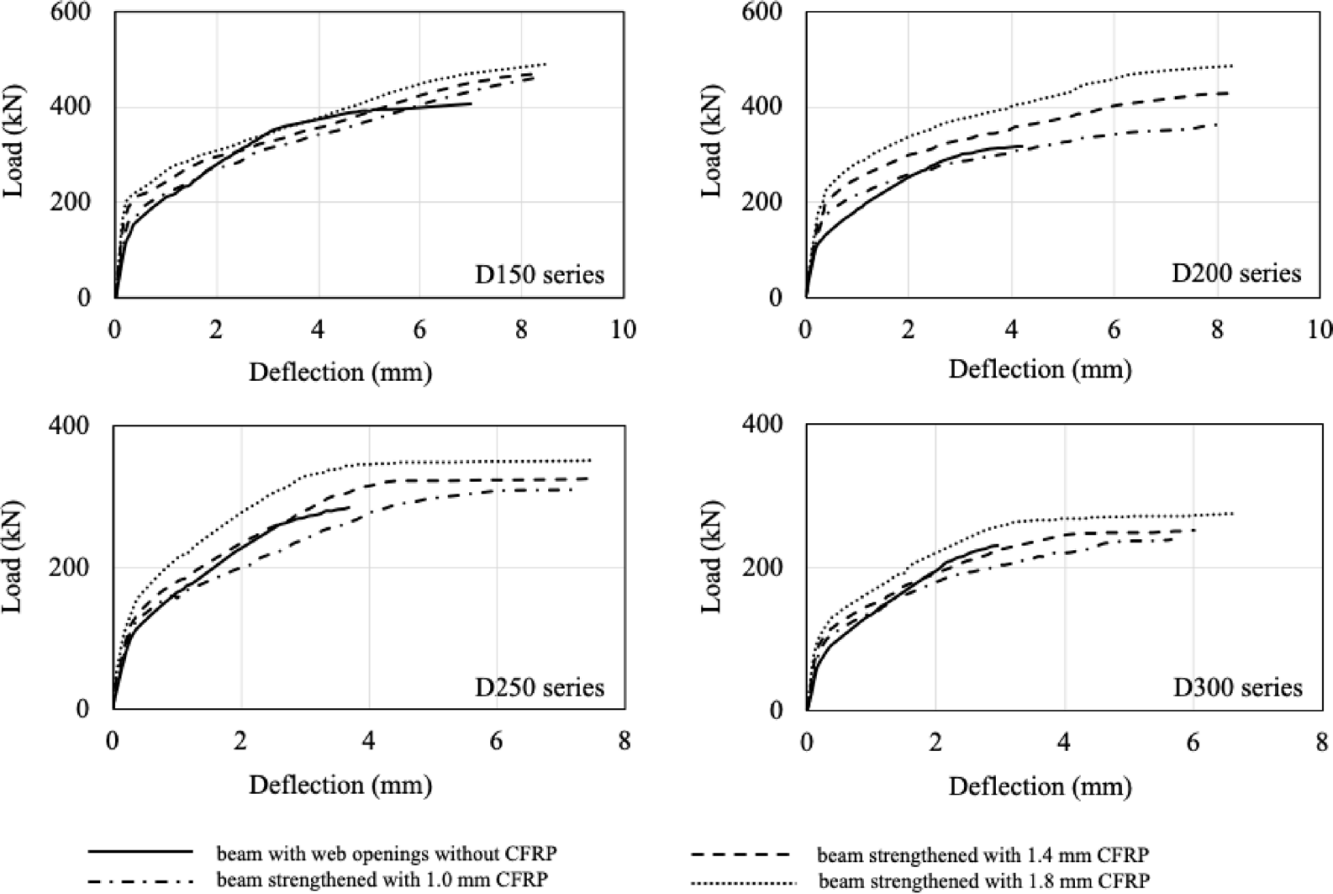
Influence of the CFRP thickness on the load-displacement relationship.

**Fig. 13:**
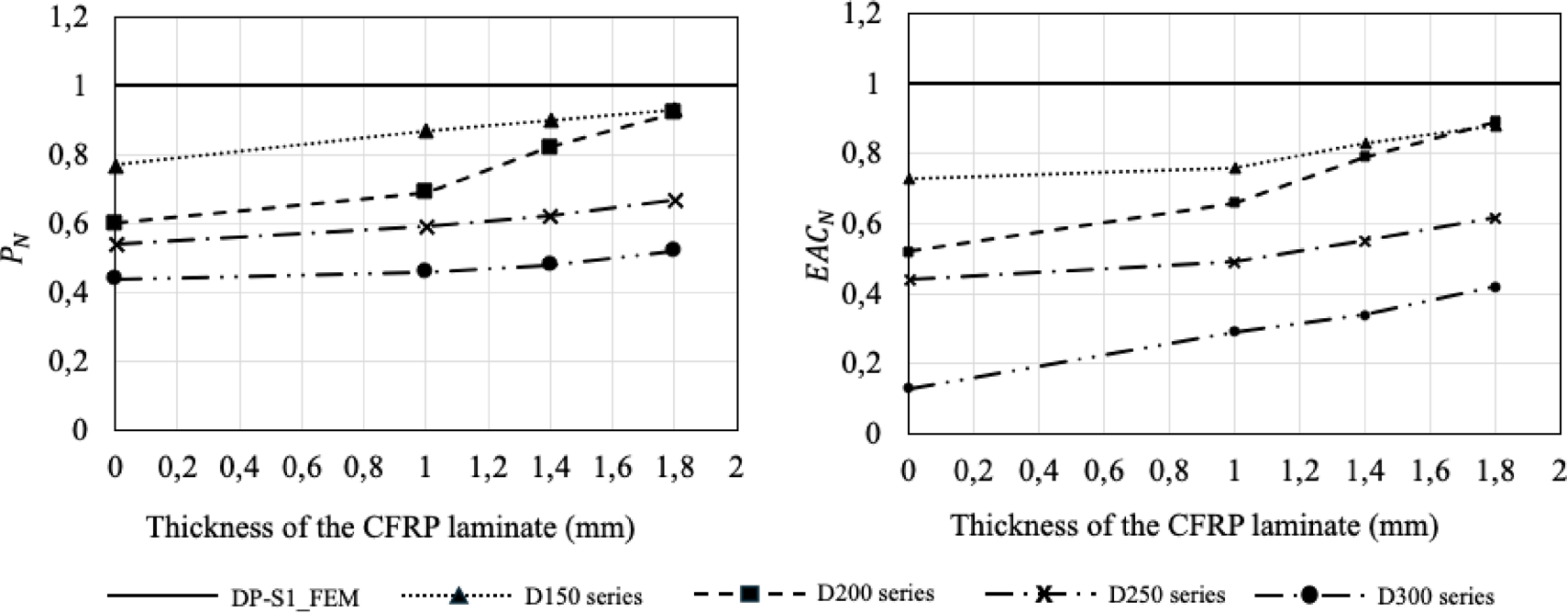
Influence of the CFRP thickness and web opening diameter on the load-carrying capacity and energy consumption capacity.

The influence of externally bonded CFRP laminates of varying thickness on the load-displacement behavior of the specimens is illustrated in Fig. 12. The application of CFRP laminates improved both the maximum load capacity and displacement ratios of the strengthened specimens. Comparative values are provided in Table 4. For all series, a slight increase in initial stiffness was observed following CFRP strengthening. Figure 13 presents a clearer comparison of the performance enhancement with respect to laminate thickness. The normalized load versus laminate thickness relationship indicates that the load capacity generally increases with increasing laminate thickness for all series; however, the rate of increase was non-uniform. For specimens strengthened with 1.0 mm laminates, the load capacity increased by 13%, 15%, 9%, and 5% for D150, D200, D250, and D300, respectively. With 1.4 mm laminates, these values were 17%, 36%, 15%, and 9%, and with 1.8 mm laminates, 21%, 53%, 24%, and 18%. It is noteworthy that even for large opening sizes, increasing laminate thickness does not significantly enhance the load-carrying capacity. For 1.8 mm laminates, however, the maximum load capacities of the 150 mm and 200 mm openings became nearly equivalent. Nonetheless, all strengthened specimens exhibited maximum loads below that of the DP-S1_FEM control specimen.

Normalized energy absorption versus laminate thickness curves (Fig. 13) reveal that energy consumption increases as the opening diameter decreases and laminate thickness increases. This increase is attributed to the combined contribution of the laminates to load capacity and displacement. Among specimens strengthened with 1.0 mm laminates, the normalized energy consumption increased by 4%, 27%, 11%, and 123% for D150, D200, D250, and D300, respectively. For 1.4 mm laminates, the corresponding values were 14%, 35%, 25%, and 161%, and for 1.8 mm laminates, 21%, 70%, 41%, and 223%. The peak stiffness values ($\:k$) presented in Table 4 were determined by dividing the $\:{P}_{U}$ by the $\:{\varDelta\:}_{U}$. The decreased stiffness of specimens with larger openings is expected. In the D300 series, all longitudinal reinforcements, except the bottom tensile and top compressive reinforcements, were cut, transforming the remaining top and bottom chords into a single-reinforced section that is more susceptible to compression failure. Consequently, the effect of CFRP strengthening on energy absorption is more pronounced in these specimens. The energy absorption of D150 and D200 specimens strengthened with 1.8 mm laminates approached similar levels. Nevertheless, the energy absorption capacities of specimens with web openings remained below that of the control specimen.

Normalized peak stiffness for different fiber volume fractions, aspect ratios, and SFRC layer thickness are presented in Table 4. Peak stiffness is computed by dividing the peak load by the corresponding displacement and normalized with the reinforced concrete beam stiffness to examine the flexural rigidity.

**Table 4:** Results of the parametric study.

Specimen	$\:{P}_{U}$ (kN)	$\:{\varDelta\:}_{U}$ (mm)	$\:{P}_{N}$ (kN)	$\:k$ (kN/mm)	$\:EAC$ (J)	$\:{EAC}_{N}$
DP-S1-FEM	525	8.50	1.00	61.76	3610.82	1.00
D150-0.0	405	7.00	0.77	57.86	2634.78	0.73
D150-1.0	460	8.27	0.87	55.62	2757.76	0.76
D150-1.4	470	8.40	0.90	55.95	2981.59	0.83
D150-1.8	488	8.45	0.93	57.75	3161.05	0.88
D200-0.0	317	4.21	0.60	75.29	1895.68	0.52
D200-1.0	365	8.20	0.69	44.51	2389.08	0.66
D200-1.4	430	8.35	0.82	51.49	2857.94	0.79
D200-1.8	485	8.42	0.92	57.60	3225.58	0.89
D250-0.0	285	3.67	0.54	77.66	1573.67	0.44
D250-1.0	310	7.29	0.59	42.52	1763.22	0.49
D250-1.4	325	7.44	0.62	43.68	2001.79	0.55
D250-1.8	351	7.49	0.67	46.86	2255.66	0.62
D300-0.0	230	2.96	0.44	77.70	464.74	0.13
D300-1.0	240	5.65	0.46	42.48	1053.21	0.29
D300-1.4	252	6.00	0.48	42.00	1226.25	0.34
D300-1.8	275	6.60	0.52	38.18	1530.71	0.42

To enable a clearer interpretation of the results, the obtained values were normalized with respect to the web opening geometry and are presented in Table 5. For each beam, the ultimate shear force $\:{V}_{U}$​ reported in the table was calculated as $\:{V}_{U}={P}_{U}/2$. In order to allow direct comparison among beams with different opening configurations and to ensure consistency with previously reported experimental studies, the results were normalized with respect to beam geometry. Accordingly, the ultimate shear capacity was expressed in terms of the nominal shear stress, $\:{v}_{U}={V}_{U}/\left({b}_{w}d\right)$.

Furthermore, to isolate the influence of concrete compressive strength, the shear stress was additionally normalized with respect to $\:\sqrt{{{f}_{c}}^{{\prime\:}}}$, yielding the parameter $\:{v}_{U}/\sqrt{{{f}_{c}}^{{\prime\:}}}$.

To quantitatively characterize the effect of the web opening geometry on the structural response, the dimensionless opening diameter ratio $\:\left(D/h\right)$ was adopted as the primary geometric parameter in this study.

To further elucidate the relative effects of the web opening and strengthening parameters, two capacity ratios were also reported. The first ratio, $\:{\eta\:}_{N}$​, represents the shear capacity of each beam normalized with respect to the solid reference beam (DP-S1-FEM). The second ratio, $\:{\eta\:}_{S}$​, compares the capacity of each strengthened beam to that of the unstrengthened beam with the same opening diameter. These normalized indicators enable a clear assessment of the influence of opening size and strengthening level on beam behavior, independent of absolute beam dimensions.

**Fig. 14:**
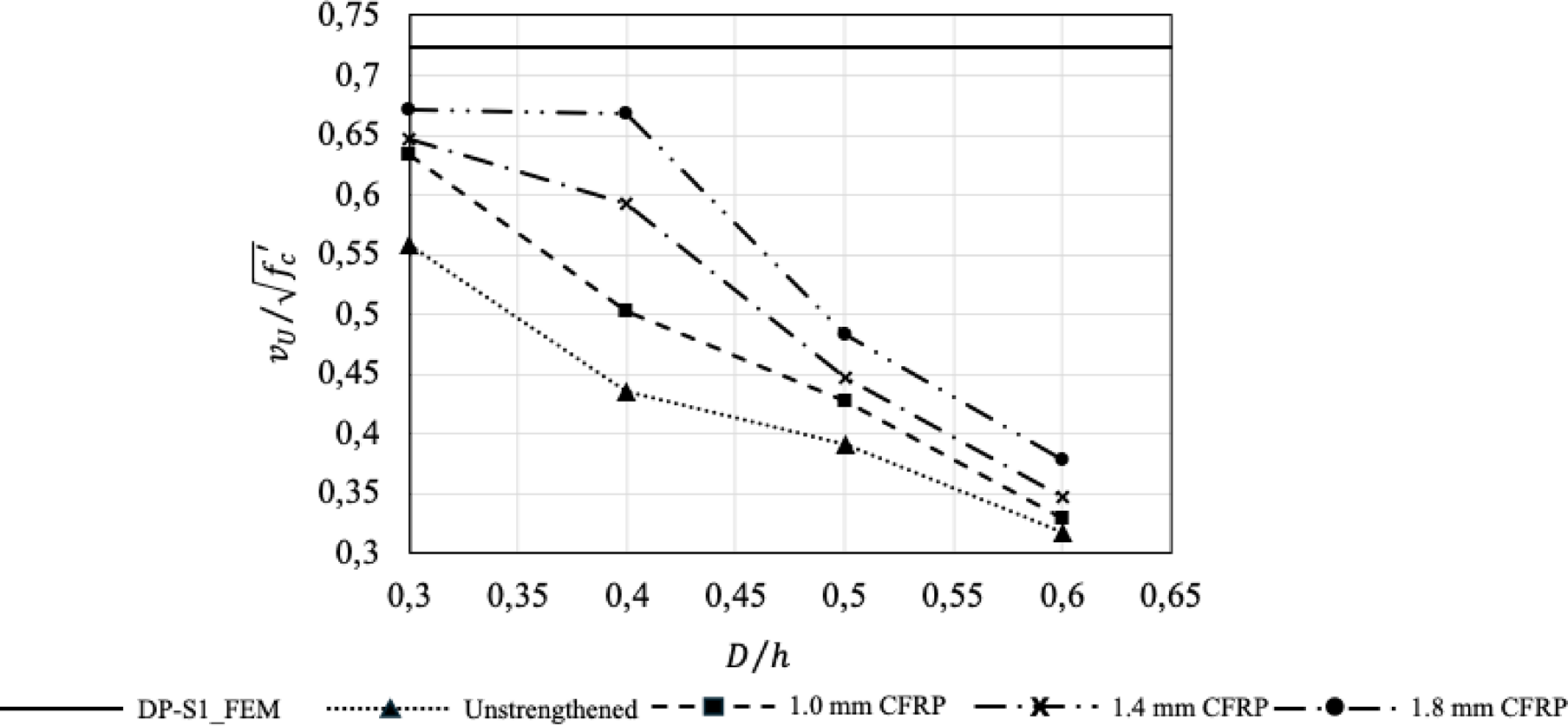
Variation of the normalized shear strength with the normalized opening diameter for different CFRP thicknesses, including comparison with the solid reference beam.

When the normalized shear strength values presented in Table 5 are evaluated together with the trends illustrated in Fig. 14, it is clearly observed that the web opening diameter has a decisive influence on the shear behavior of reinforced concrete deep beams. As the opening diameter increases, a systematic reduction in normalized shear strength is observed for all CFRP thicknesses, indicating that opening geometry is the dominant parameter governing the shear capacity. Beams with smaller opening diameters (D150 and D200 series) exhibit relatively higher normalized shear strength, whereas a pronounced capacity reduction is observed for the D250 and, in particular, the D300 series as the opening size increases.

For all series, increasing the CFRP laminate thickness leads to an improvement in normalized shear strength; however, the relative effectiveness of this improvement decreases with increasing opening diameter. This observation indicates that CFRP strengthening is more effective in enhancing shear capacity for beams with small to moderate openings, whereas for larger openings it can only partially compensate for the capacity loss induced by the presence of the opening. The comparison with the solid reference beam further confirms that, despite strengthening, web openings impose a persistent adverse effect on shear capacity.

With increasing opening diameter, the associated increase in stirrup damage is expected to promote the concentration of shear cracks around the opening and to reduce the contribution of stirrups to the shear-resisting mechanism. In particular, for large opening diameters, inclined shear cracks are more likely to intersect or overstress the stirrup reinforcement at earlier stages, resulting in a more brittle shear response. Although stirrup damage was not explicitly quantified in this study, the progressive reduction in stirrup effectiveness with increasing opening size may be regarded as a secondary mechanism contributing to the observed decrease in normalized shear strength.

Overall, these results demonstrate that the use of normalized parameters provides a clear and consistent framework for assessing the combined effects of web opening diameter and CFRP laminate thickness on the shear behavior of reinforced concrete deep beams.

The findings obtained in this study were also evaluated using the design provisions of the Concrete Society TR55 guideline^56^. In this context, the shear capacities calculated in accordance with the TR55 provisions $\:\left({V}_{TR55}\right)$ and their ratios to the shear capacities obtained from the finite element analyses, $\:{V}_{U}$, expressed as

$\:{V}_{TR55}/{V}_{U}$, are presented in Table 5.

The CFRP strengthening system was idealized as being applied to both side faces of the beam without the use of mechanical anchorage. The CFRP fibers were assumed to be oriented perpendicular to the beam axis so as to directly intersect potential diagonal crack planes $\:(\alpha\:=90^\circ\:)$. For externally bonded CFRP systems, the effective CFRP strain was fixed at $\:{\epsilon\:}_{fe}=0.004$, consistent with the upper-bound value commonly adopted in the literature. The internal lever arm was defined as $\:{z}_{f}=0.9h$, based on the total beam depth of h = 500 mm.

An examination of the $\:{V}_{TR55}/{V}_{U}$ ratios presented in Table 5 indicates that, for the beam configurations considered within the scope of this study, the shear capacities predicted using the TR55 design approach exhibit a generally comparable trend to those obtained from the finite element analyses. For specimens with small to moderate web opening sizes (D150 and D200 series), the fact that the $\:{V}_{TR55}/{V}_{U}$ ratios are predominantly greater than 1.0 indicates that the TR55 approach predicts higher shear capacities than those obtained from the FEM results for these configurations. In contrast, the concentration of the ratios around 1.0 for the D250 series indicates a good level of agreement between the TR55 predictions and the FEM results at this opening level. For the largest opening size considered (D300 series), the reduction of the $\:{V}_{TR55}/{V}_{U}$ ratios below 1.0 indicates that, in these critical geometries where the primary compression load-transfer path is significantly weakened, the shear capacities predicted by TR55 are lower than those obtained from the finite element analyses. Nevertheless, given that these comparisons are based on the properties of a single experimental reference beam, the findings should be interpreted within the scope of the present study, and further investigations involving different opening geometries, reinforcement layouts, and beam sizes would be beneficial to support broader conclusions.

**Table 5:** Normalized shear strength values and comparison of TR55 design provisions with FEM results.

Specimen	$\:D/h$	$\:{V}_{U}$ (kN)	$\:{v}_{U}$ (MPa)	$\:{v}_{U}/\sqrt{{{f}_{c}}^{{\prime\:}}}$	$\:{\eta\:}_{N}$	$\:{\eta\:}_{S}$	$\:{V}_{TR55}$	$\:{V}_{TR55}/{V}_{U}$
DP-S1-FEM	-	262.5	3.755	0.723	1.000	-	-	-
D150-0.0	0.30	202.5	2.897	0.558	0.771	1.000	-	-
D150-1.0	0.30	230.0	3.290	0.633	0.876	1.136	211.7	1.09
D150-1.4	0.30	235.0	3.362	0.647	0.895	1.160	220.6	1.07
D150-1.8	0.30	244.0	3.491	0.672	0.930	1.205	232.4	1.05
D200-0.0	0.40	158.5	2.268	0.436	0.604	1.000	-	-
D200-1.0	0.40	182.5	2.611	0.502	0.695	1.151	167.7	1.09
D200-1.4	0.40	215.0	3.076	0.592	0.819	1.356	176.6	1.22
D200-1.8	0.40	242.5	3.469	0.668	0.924	1.530	188.4	1.29
D250-0.0	0.50	142.5	2.039	0.392	0.543	1.000	-	-
D250-1.0	0.50	155.0	2.217	0.427	0.590	1.088	151.7	1.02
D250-1.4	0.50	162.5	2.325	0.447	0.619	1.140	160.6	1.01
D250-1.8	0.50	175.5	2.511	0.483	0.669	1.232	172.4	1.02
D300-0.0	0.60	115.0	1.645	0.317	0.438	1.000	-	-
D300-1.0	0.60	120.0	1.717	0.330	0.457	1.043	124.2	0.97
D300-1.4	0.60	126.0	1.803	0.347	0.480	1.096	133.1	0.95
D300-1.8	0.60	137.5	1.967	0.379	0.524	1.196	144.9	0.95

## Conclusions

This study investigates the structural behavior of reinforced concrete deep beams containing post-installed circular web openings formed by cutting the stirrup reinforcement within the shear span. Unlike openings that are planned and properly detailed during the design stage, such interventions directly disrupt the shear load-transfer mechanism and the continuity of internal force paths, thereby significantly affecting beam behavior. Within this framework, a finite element–based parametric approach was employed to systematically evaluate the effects of opening diameter and CFRP laminate thickness on the load-carrying capacity, deformation behavior, and failure mechanisms of deep beams. The main findings of the study are summarized as follows:

- The analysis results indicate that finite element models are suitable for evaluating the structural response of reinforced concrete deep beams. Comparisons with the experimental reference beam demonstrated that the FEM models successfully predicted the load–displacement response, crack development, and failure modes.
- It was found that post-installed web openings created by cutting the stirrup reinforcement adversely affect structural performance compared to beams with openings planned at the design stage. For the investigated configuration, beams with cut stirrups exhibited an approximately 11.5% reduction in load-carrying capacity and about a 5% reduction in maximum displacement.
- An increase in opening diameter led to a pronounced change in the governing damage mechanism. While shear damage was dominant for smaller openings, larger openings resulted in a combined shear–compression failure mechanism, indicating that opening size influences not only the magnitude of strength reduction but also the dominant failure mode.
- For unstrengthened beams, increasing the opening diameter from 150 mm to 300 mm resulted in reductions in load-carrying capacity of 23%, 40%, 46%, and 56%, respectively. Correspondingly, the energy absorption capacity decreased by 27, 48, 56, and 87%. These results highlight the critical influence of web openings located within the shear span on the structural behavior of deep beams.
- The application of externally bonded CFRP laminates enhanced both the load-carrying capacity and energy absorption capacity of beams containing web openings. However, for all CFRP laminate thicknesses considered (1.0, 1.4, and 1.8 mm), the maximum load capacities of the strengthened beams remained lower than that of the solid reference beam.
- For larger opening diameters, increasing the CFRP laminate thickness resulted in diminishing gains in load-carrying capacity. In particular, when the opening diameter exceeded half of the beam depth, increasing the CFRP thickness to 1.8 mm yielded load-carrying and energy absorption capacities of only about 50% of those of the solid beam.
- When stirrup reinforcement located along the primary shear force flow path is cut due to post-installed web openings, the continuity of shear reinforcement is disrupted and the internal load-transfer mechanism is significantly weakened. Under these conditions, although CFRP strengthening improves structural performance, the original load-carrying capacity of the member could not be fully restored for the investigated beam geometry and opening configurations.

## Data Availability

The data that support the findings of this study are available from the corresponding author upon reasonable request.
